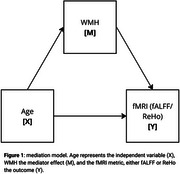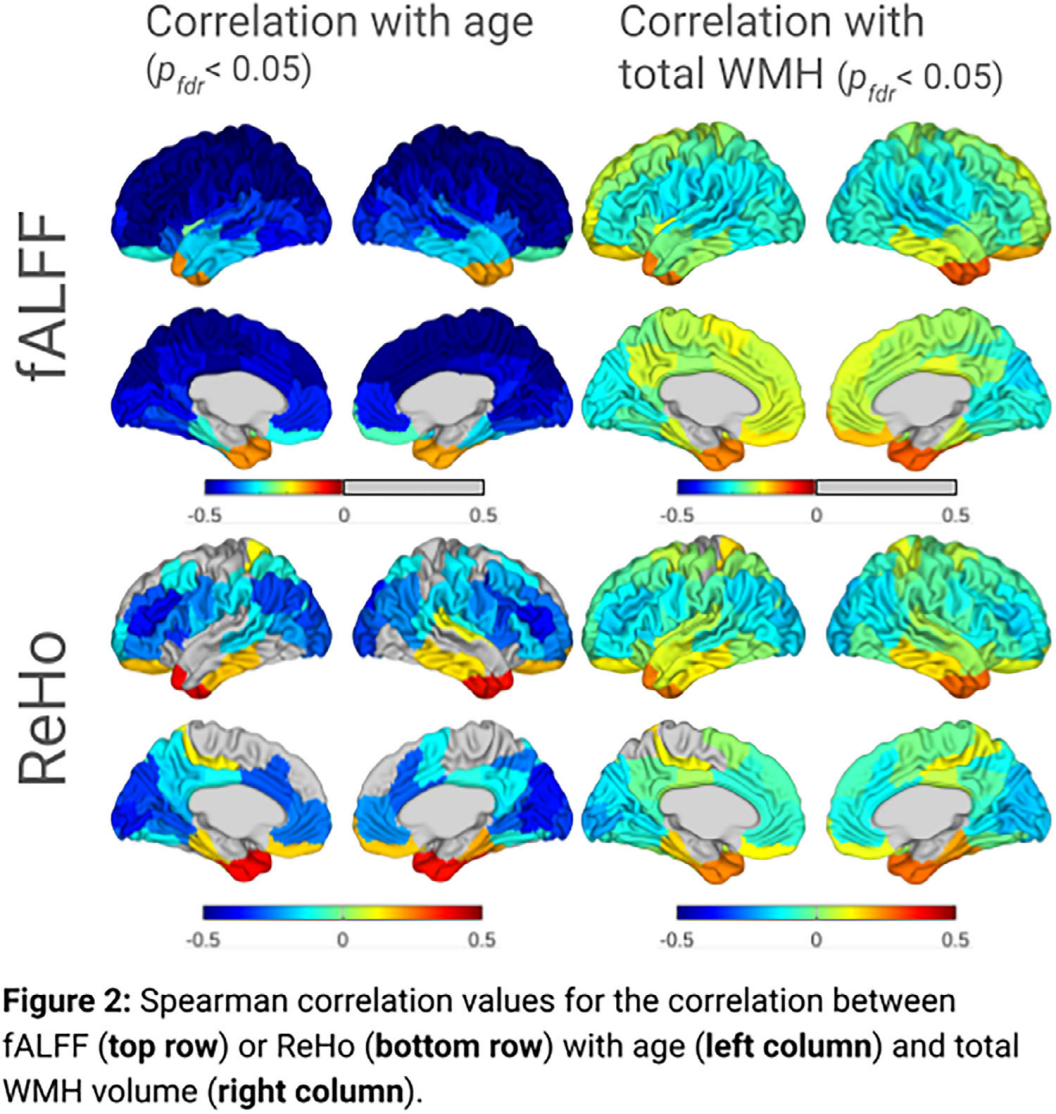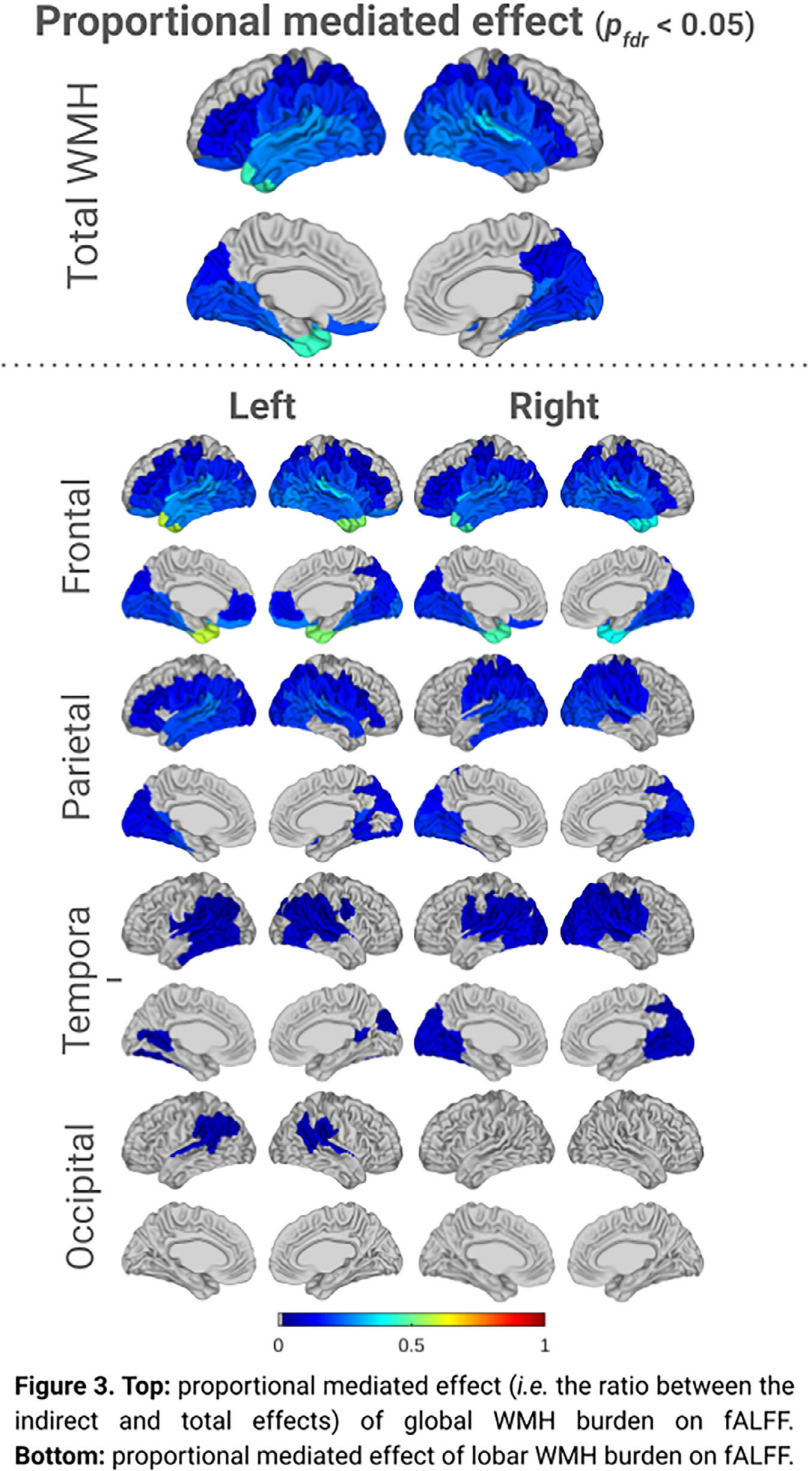# The mediating effect of white matter hyperintensities on spontaneous brain activity during aging

**DOI:** 10.1002/alz70856_105141

**Published:** 2026-01-09

**Authors:** Aliza Brzezinski‐Rittner, Amelie Metz, Roqaie Moqadam, Mahsa Dadar, Yashar Zeighami

**Affiliations:** ^1^ McGill University, Montréal, QC, Canada; ^2^ Douglas Mental Health University Institute, Montréal, QC, Canada; ^3^ Department of Psychiatry, McGill University, Montréal, QC, Canada; ^4^ University of Montreal, Montréal, QC, Canada; ^5^ Institut Universitaire de Gériatrie de Montréal, Montréal, QC, Canada; ^6^ Montreal Neurological Institute, McGill University, Montréal, QC, Canada

## Abstract

**Background:**

Aging is associated with alterations in brain functional activity observed through resting‐state functional Magnetic Resonance Imaging (rs‐fMRI, Guan et al., 2024). White matter hyperintensities (WMH), indicative of cerebral small vessel disease, are also prevalent in older adults (Hachinski et al., 1987). This study examined whether WMH burden mediates the relationship between healthy aging and intrinsic neuronal activity.

**Method:**

MRI data from 557 participants (51.7% female), aged 18 to 87 years, were obtained from the Cambridge Centre for Ageing and Neuroscience (Cam‐CAN) dataset. Total and lobar white matter hyperintensity (WMH) volumes were quantified from T1‐weighted and T2‐weighted structural MRI images (Dadar & Collins 2021), while regional fractional amplitude of low‐frequency fluctuations (fALFF) and Regional Homogeneity (ReHo) metrics were derived from rs‐fMRI using the Schaefer parcellation (Esteban et al. 2019). Spearman correlation analyses were conducted to assess the relationships between age, WMH load, and regional functional metrics. Mediation analysis was subsequently performed to investigate whether WMH burden mediated the effect of age on spontaneous neural activity (Figure 1). All results were adjusted for multiple comparisons using FDR correction.

**Result:**

fALFF exhibited significant (*p_fdr_* < 0.05) negative correlations with both age and WMHs across the cortex, while ReHo showed region‐specific correlations: positive in frontal and occipital regions and negative in the temporal pole (Figure 2). Mediation analysis revealed statistically significant (*p_fdr_* < 0.05) effects of global WMH load on the impact of age on fALFF, predominantly in occipital and temporal regions, with the strongest effect in the left temporal pole. The mediation pattern of frontal WMH burden mirrored that of global WMHs. Parietal and temporal WMHs demonstrated significant but weaker mediation effects, both in magnitude and spatial extent. Conversely, occipital WMHs showed only localized and modest effects (Figure 3). Unlike fALFF, WMHs did not significantly mediate the age‐related changes in regional ReHo.

**Conclusion:**

Our findings indicate that the impact of aging on spontaneous neural activity, specifically fALFF, is mediated by WMH burden. The mediation effect is pronounced for frontal WMHs, often attributed to vascular origin (Pålhaugen et al., 2020), suggesting a potential link between vascular processes and age‐related changes in neural activity.